# The association between blood urea nitrogen to serum albumin ratio and 28 day in-hospital mortality in patients with chronic heart failure and sepsis: a pilot retrospective study

**DOI:** 10.3389/fcvm.2025.1491331

**Published:** 2025-03-04

**Authors:** Ali Ma, Chen Zhang, Ying Gong, Xueping Ma, Ning Yan

**Affiliations:** ^1^ First Clinical College, Ningxia Medical University, Yinchuan, China; ^2^ Heart Centre & Department of Cardiovascular Diseases, General Hospital of Ningxia Medical University, Yinchuan, China; ^3^ Institute of Medical Sciences, General Hospital of Ningxia Medical University, Yinchuan, China

**Keywords:** blood urea nitrogen to serum albumin ratio, congestive heart failure, sepsis, eICU, in-hospital mortality

## Abstract

**Aims:**

The purpose of this study was to explore the relationship between blood urea nitrogen to serum albumin ratio and 28-day in-hospital mortality in patients with chronic heart failure complicated by sepsis admitted to the intensive care unit (ICU).

**Methods:**

This retrospective study included 723 patients with chronic heart failure complicated by sepsis from the eICU database. Smooth curve fitting assessed the association between BAR and mortality. Multivariable Cox regression analysis was conducted to calculate the adjusted hazard ratio (HR) and 95% confidence interval (CI). Kaplan–Meier curves compared survival rates across BAR tertile*s*. Subgroup analysis was stratified based on relevant covariates and a forest plot was drawn to verify the stability of the results.

**Results:**

Among 723 chronic heart failure patients with sepsis, the 28-day mortality rate was 20.33% (147/723). After adjusting for confounders, with BAR as a categorical variable, patients in the highest tertile of BAR had a significantly higher death risk than those in the lowest tertile [HR: 1.87, 95% CI (1.09,3.19), *p*: 0.023]. When BAR was a continuous variable, each unit increase in BAR raised in—hospital mortality by 2% [HR: 1.02, 95% CI (1.01, 1.04), *p* = 0.0038]. Stratified analysis showed no interaction, and E—value analysis indicated robustness to unmeasured confounding, highlighting the stable and significant relationship between BAR and 28—day mortality in these patients.

**Conclusion:**

In the context of critically ill patients with chronic heart failure complicated by sepsis, there exists a significant correlation between blood urea nitrogen to serum albumin ratio (BAR) and 28-day mortality. Specifically, higher BAR levels are associated with an elevated risk of 28-day mortality in these patients. However, these findings require further research for confirmation.

## Introduction

Chronic heart failure (CHF) refers to a clinical syndrome where the heart, due to structural or functional abnormalities, is unable to pump blood effectively to meet the metabolic demands of the entire body's tissues. This clinical syndrome not only involves cardiac dysfunction itself but may also affect the health status of multiple organs, thereby leading to a series of complex pathophysiological changes (1). According to global statistics, the incidence of chronic heart failure is between 1% and 2%, and this proportion increases significantly with the growth of the population's age. Among the elderly population over 60 years old, the incidence rate can reach 6% to 10%. Even more worryingly, chronic heart failure has become one of the major public health problems worldwide, ranking high among the causes of death, with a mortality rate of approximately 50% within 5 years. Currently, because of population growth and the aging of society, the total number of patients with heart failure is still on the rise. Similarly, there is evidence suggesting that the number of patients with heart failure may be increasing in low-income countries struggling under the double burden of infectious diseases and diseases related to the Western lifestyle. These findings, coupled with the slower decline in the mortality rate of heart failure than previously observed, indicate that we have not yet reached the end of this epidemic (2–4). Currently, there are various types of drugs for the treatment of chronic heart failure in the world, such as angiotensin receptor-neprilysin inhibitors, ACE inhibitors, angiotensin II receptor antagonists, beta-blockers, aldosterone antagonists, sodium-glucose cotransporter 2 inhibitors, soluble guanylate cyclase stimulators, ivabradine, digitalis drugs, cardiac resynchronization therapy, and implantable cardioverter-defibrillators, etc. Despite increasingly novel treatment regimens, the mortality rate and rehospitalization rate of chronic heart failure remains high (5, 6).

The Sepsis-3 Consensus defines sepsis as “organ dysfunction caused by a dysregulated host response to infection”, highlighting the critical role of immune dysregulation (7). Approximately 49 million people are affected by sepsis each year, and it is estimated that this syndrome causes 11 million deaths, accounting for 19.7% of global deaths. Globally, the average mortality rate seems to be declining, but still, up to 25% of patients die from sepsis. The guidelines explicitly aim to immediately initiate fluid resuscitation and sepsis management measures (8). The “Hour-1-Bundle” includes 5 clinical intervention measures: blood culture before antibiotics, administration of broad-spectrum antibiotics, administration of intravenous fluids, application of vasopressors, and measurement of lactate levels (9).

The impact of sepsis on the heart is multi-faceted, including increased cardiac load, myocardial depression, and electrophysiological changes, and patients with chronic heart failure are more prone to disease deterioration when dealing with sepsis. On the one hand, chronic heart failure may lead to a decreased tolerance of the body, making the inflammatory response triggered by sepsis more severe; on the other hand, the progression of sepsis may aggravate the symptoms and course of heart failure, forming a vicious cycle (10–12). In critical care medicine, patients with chronic heart failure and sepsis face a high mortality rate. Identifying effective prognostic markers is crucial. Serum albumin and blood urea nitrogen levels are closely linked to patient mortality and immune status (13). Hypoalbuminemia, common in these patients, indicates poor nutrition and weakens immunity, raising the death risk, with sepsis—related inflammation further lowering albumin (14). Elevated blood urea nitrogen reflects kidney damage from heart failure and sepsis—induced issues, disrupting immunity and increasing mortality (15). In recent years, the ratio of blood urea nitrogen to serum albumin (BAR) has emerged as a new prognostic biomarker, representing an index of nutritional status and inflammatory levels. Extensive studies have investigated the predictive value of BAR for various diseases, and BAR has been used to assess mortality and disease severity in patients with community-acquired pneumonia. Notably, BAR has been identified as an independent predictor of mortality and the need for intensive care in patients with community-acquired pneumonia, indicating its potential in predicting the prognosis of critically ill patients (16). Additionally, elevated BAR shows significant prognostic value in patients with acute exacerbation of chronic obstructive pulmonary disease (AECOPD) and heart failure (17). In particular, elevated BAR index is associated with increased mortality in patients with coronary heart disease in the intensive care unit, suggesting its role in the early identification of high-risk patients and guiding active clinical management (18). Based on the above findings, therefore, how to manage patients with sepsis and chronic heart failure in clinical practice has become an important issue that urgently needs to be addressed, but currently, there are still relatively few early warning indicators related to the death of patients with chronic heart failure and sepsis. To search for readily available biomarkers to determine the prognosis of patients with chronic heart failure and sepsis, we considered BAR. Currently, the relationship between BAR and the prognosis of patients with severe chronic heart failure and sepsis remains unclear.

Based on the above, the objective of this study is to investigate the correlation between BAR and the prognosis of patients with chronic heart failure and sepsis in the ICU. The intention is to offer valuable insights into the clinical management of this disease and furnish a scientific foundation and practical guidance for enhancing patient prognosis.

## Material and method

### Data source

This retrospective observational study utilized data extracted from the eICU Collaborative Research Database (eICU-CRD), an online international database. The eICU-CRD is a multicenter ICU database containing highly detailed data from over 200,000 ICU admissions monitored by the eICU program across the United States. Data from 2014 to 2015 were automatically stored and electronically retrieved through the Philips Healthcare eICU program. The eICU-CRD has been extensively used for observational research. Access to this database requires passing an examination and obtaining certification as per the data usage agreement of the PhysioNet Review Board. This database operates under the Health Insurance Portability and Accountability Act (HIPAA) Safe Harbor provision. Approval to access the data was granted after completing the Collaborative Institutional Training Initiative (CITI) program for “Data or Specimens Only Research”. Due to its retrospective design, lack of direct patient intervention, and certified security schema meeting safe harbor standards by Privacert (Cambridge, MA), this study was exempted from approval by the Institutional Review Board of the Massachusetts Institute of Technology (our record ID: 13574411). Informed consent was waived for the same reasons. The study adhered to the principles of the Declaration of Helsinki, and all methodologies were conducted in compliance with relevant guidelines and regulations.

### Population

**Figure 1:**
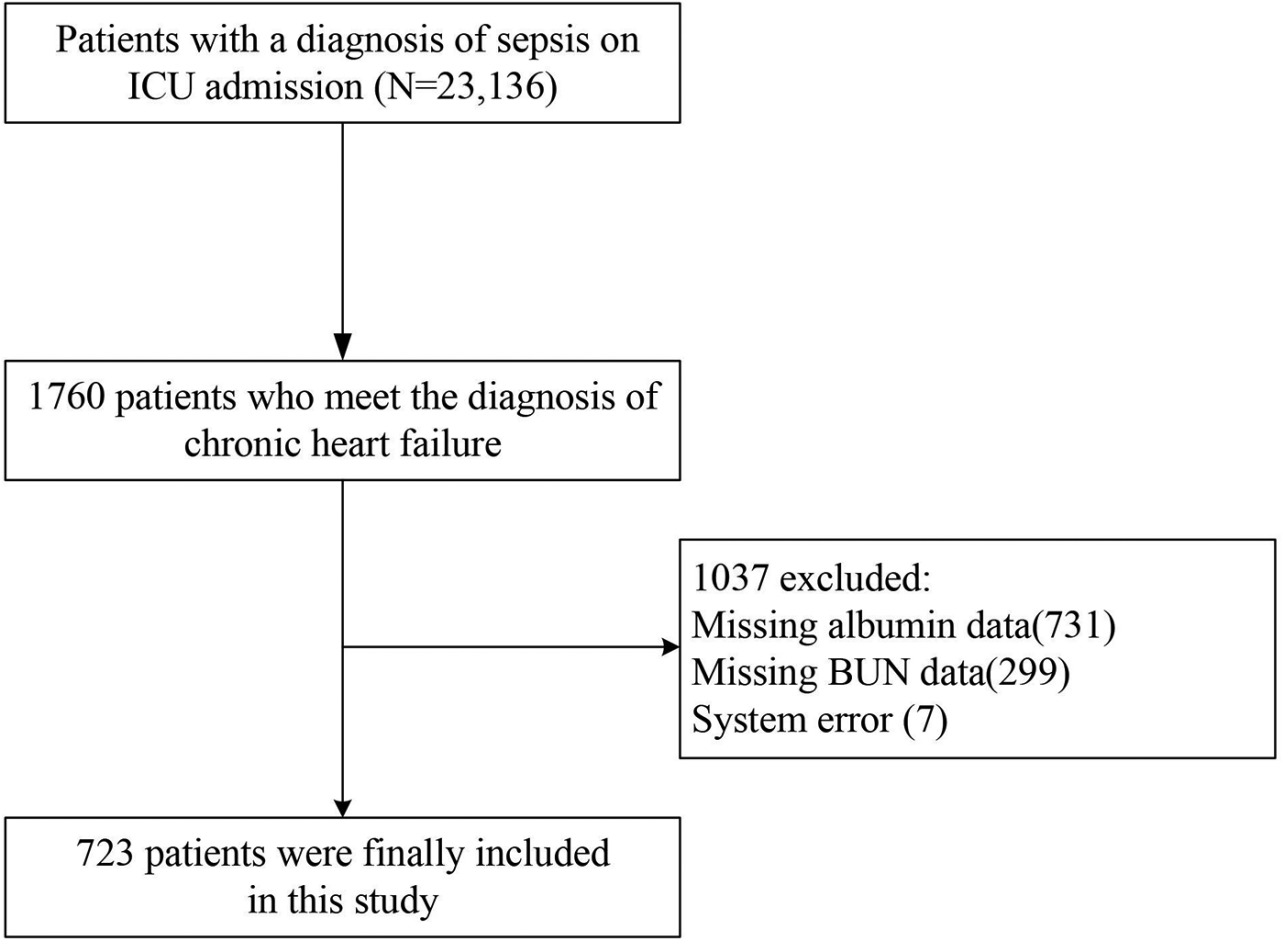
Flowchart.

In the study population, patients admitted to the intensive care unit (ICU) with a diagnosis of sepsis and chronic heart failure, sepsis was defined according to Sepsis-3.0 criteria (2016) as an increase of ≥2 points in the Sequential Organ Failure Assessment (SOFA) score plus confirmed or suspected infection (19), and the diagnosis of chronic heart failure was based on the patient's clinical presentation, echocardiography showing structural and functional abnormalities of the heart (e.g., decreased ejection fraction), and blood testing for BNP or NT-proBNP (20, 21). Sepsis and chronic heart failure were identified according to the ICD-9 code in the eICU Cooperative Research Database. The selection process of the study population was as follows: 23,136 individuals met the diagnostic criteria for sepsis. Out of these, 1,760 individuals met the diagnostic criteria for chronic heart failure. Additionally, 731 individuals were missing albumin data, leaving 1,029 individuals. Furthermore, 299 individuals were missing BUN data, resulting in 723 individuals who were finally included in the study, as shown in Figure 1.

### Variates

Data collected within 24 h of ICU admission was extracted from eICU-CRD. Based on previous studies, clinical experience, and univariate analysis of *p* < 0.05 variables, we used the following data as confounding variables for the prognosis of patients with sepsis and chronic heart failure, including: Physiological indicators: Gender, Age (years), BMI (Kg/m^2^), Heart rate (/min), MAP (mmHg), Laboratory tests: AST aspartate transaminase (U/L), total protein (g/dl), Related Scoring System & Disease: Arrhythmia, Diabetes, SOFA score, GCS score.

### Primary outcomes

In this study, “28-day mortality” is defined as the occurrence of death from any cause within 28 days following the initiation of hospitalization. This metric serves as a critical endpoint for evaluating patient outcomes and the effectiveness of interventions in a clinical setting. During this 28-day period, we closely monitored all patients admitted to the hospital, meticulously tracking and recording all instances of mortality, regardless of the underlying causes-be it related to the primary condition for which the patient was hospitalized, co-morbid conditions, unexpected complications, or unrelated health issues. Data were obtained through thorough examination of medical records, consultation with clinical staff, and relevant health registries to ensure accurate identification and confirmation of each death event.

### Statistical analysis

BAR is calculated from blood urea nitrogen and albumin. Descriptive analysis of variables is conducted using the Kruskal Wallis rank sum test with BAR tertiles [Low BAR (1.2–9.72), Middle BAR (10.0–17.93), High BAR (18.1–56.88)]. Continuous variables are described as median (Q1, Q3), and categorical variables are presented in the form of quantity and percentage. Smooth curve fitting was used to analyze the linear relationship between BAR and 28-day mortality risk in patients by adjusting for all covariates in Model III. Three multivariable Cox proportional hazard models were constructed to evaluate the independent association between BAR and in-hospital mortality, Adjust I model adjusts for Gender, Age (years). Adjust II model adjusts for: Gender, Age (years), BMI (Kg/m^2^), COPD, Diabetes, Arrhythmia, MAP(mmHg), Heart rate (/min), Temperature, AST(U/L), total protein (g/dl). Adjust III model adjusts for: Gender, Age (years), BMI (Kg/m^2^), COPD, Diabetes, Arrhythmia, MAP (mmHg), Heart rate (/min), Temperature, AST (U/L), total protein (g/dl), SOFA score, GCS score. Stratification and interaction analyses are performed based on GENDER (male or female), AGE (>65 years old or ≤65 years old), COPD (yes or no), DIABETES (yes or no), Arrhythmia (yes or no), SOFA score (≤5 points or >5 points), GCS score (≤11 points or >11 points). Survival curves are estimated using the Kaplan–Meier method and compared by the log-rank test. All statistical analyses are performed using EmpowerStats (www.empowerstats.com, X&Ysolutions, Inc. Boston MA) and R software version 3.6.1 (http://www.r-project.org).

### Sensitivity analysis

We explored the potential for unmeasured confounding between BAR and 28-day in-hospital mortality by calculating E-values. The E-value quantifies the required magnitude of an unmeasured confounder that could negate the observed association between BAR and 28-day in-hospital mortality. This approach allows us to better understand the robustness of our findings and the reliability of BAR as a potential biomarker for predicting the prognosis of patients with chronic heart failure and sepsis (22). In order to more effectively verify the association between BAR and 28 day mortality, propensity score matching (PSM) sensitivity analysis was conducted, and 342 subjects were included after matching. Multivariate Cox regression analysis was then performed to examine the relationship between BAR and 28 day mortality.

## Results

### Baseline characteristics of participants and outcome parameters

The basic demographic characteristics of all selected patients stratified by bar tertiles are shown in Table 1. The median age of the total population was 73 years, 364 men (50.35%), and the 28 day all-cause mortality rate was 20.33% (147/723). The results of grouping according to the tertile of BAR showed that the 28 day all-cause mortality of T1 group was 10.04% (24 cases), that of T2 group was 23.24% (56 cases), and that of T3 group was 27.57% (67 cases), among which the mortality of T3 group was significantly higher. The age, SOFA score, acute physiology score III, Apache IV score, blood urea nitrogen and serum creatinine of T3 group were higher, while albumin, total protein, heart rate and map were lower.

**Table 1:** Baseline characteristics of participants and outcome parameters.

Variables	Total	BAR			*p*-value
		T1 (1.2–9.72)	T2 (10.0–17.93)	T3 (18.0–56.88)	
Participants	723	239	241	243	
Age	73.00 (63.00–82.00)	71.00 (61.00–79.00)	74.00 (65.00–84.00)	74.00 (63.00–83.00)	0.005
BMI	27.90 (23.30–34.12)	28.30 (23.30–34.22)	27.37 (22.58–33.35)	28.12 (23.78–34.73)	0.676
Gender					0.094
Male	364 (50.35%)	130 (54.39%)	125 (51.87%)	109 (44.86%)	
Female	359 (49.65%)	109 (45.61%)	116 (48.13%)	134 (55.14%)	
Score system					
GCS score	14.00 (11.00–15.00)	15.00 (12.00–15.00)	14.00 (11.00–15.00)	14.00 (10.00–15.00)	0.16
SOFA score	5.00 (2.00–7.00)	3.00 (2.00–5.50)	5.00 (3.00–7.00)	6.00 (4.00–8.00)	<0.001
Acute Physiology Score III	55.00 (43.50–72.50)	47.00 (35.00–61.00)	55.00 (45.00–70.00)	66.00 (53.00–82.50)	<0.001
Apache IV score	73.00 (58.00–89.50)	61.00 (50.00–78.00)	73.00 (60.00–88.00)	82.00 (70.00–98.00)	<0.001
Vital signs					
MAP	55.00 (46.00–118.50)	60.00 (48.00–125.50)	55.00 (47.00–116.00)	52.00 (44.50–71.00)	0.021
Heartrate	110.00 (93.00–128.00)	114.00 (98.50–132.50)	108.00 (93.00–125.00)	108.00 (89.00–126.50)	0.041
Temperature	36.40 (36.10–36.80)	36.50 (36.20–36.90)	36.40 (36.05–36.70)	36.40 (35.90–36.70)	<0.001
Laboratory data					
BUN (mg/dl)	35.00 (23.00–53.00)	19.00 (15.00–23.50)	36.00 (30.00–43.00)	64.00 (50.00–80.00)	<0.001
Scr(mg/dl)	1.69 (1.10–2.66)	1.10 (0.81–1.40)	1.70 (1.20–2.30)	2.70 (1.90–3.79)	<0.001
AST(U/L)	38.00 (22.00–76.00)	30.00 (20.00–57.50)	39.00 (23.00–96.00)	44.00 (22.00–108.50)	0.667
Total protein (g/dl)	5.90 (5.40–6.50)	6.20 (5.60–6.70)	6.00 (5.40–6.50)	5.70 (5.15–6.40)	<0.001
Albumin (g/dl)	2.70 (2.30–3.10)	2.90 (2.50–3.20)	2.70 (2.30–3.10)	2.40 (2.10–2.80)	<0.001
Comorbidity disease					
COPD	153 (21.16%)	62 (25.94%)	52 (21.58%)	39 (16.05%)	0.029
AMI	61 (8.44%)	19 (7.95%)	25 (10.37%)	17 (7.00%)	0.387
Diabetes	212 (29.32%)	55 (23.01%)	78 (32.37%)	79 (32.51%)	0.032
Pneumonia	353 (48.82%)	128 (53.56%)	109 (45.23%)	116 (47.74%)	0.173
Arrhythmia	265 (36.65%)	83 (34.73%)	82 (34.02%)	100 (41.15%)	0.2
Primary outcomes					
28-day all-cause mortality	147 (20.33%)	24 (10.04%)	56 (23.24%)	67 (27.57%)	<0.001

GCS score, glasgow coma scale score; SOFA score, sequential organ failure assessment score; BUN, blood urea nitrogen (mg/dl); Scr, Serum creatinine (mg/dl); AST, aspartate transaminase (U/L); COPD, chronic obstructive pulmonary disease; AMI, acute myocardial infarction; MAP, mean arterial pressure (mmHg).

### The relationship between BAR and 28-day mortality

**Figure 2:**
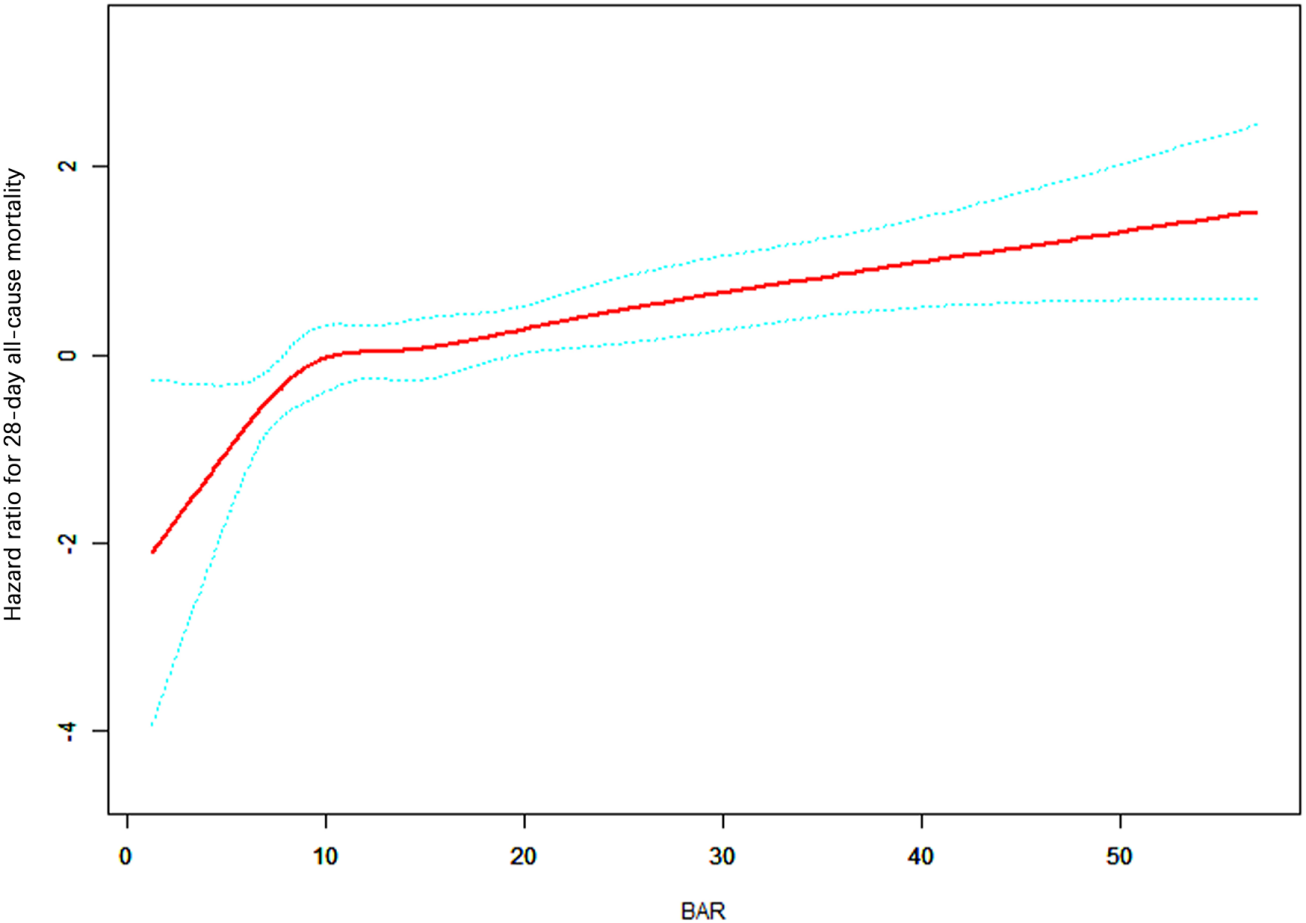
Smoothed curve—fitting graph.

In this study, we used a smoothed curve fitting method to analyze the linear relationship between BAR and a patient's 28-day risk of all-cause death. Specifically, the red curve represents the 28-day risk of all-cause death, while the blue curve represents its 95% confidence interval. We found a significant non-linear relationship between BAR and 28-day all-cause mortality through threshold effect analysis (Figure 2 and Table 2). In Model II, after adjusting for gender, age, BMI, COPD, diabetes, arrhythmia, mean arterial pressure, heartrate, temperature, AST, total protein, SOFA score, and GCS score, the turning point (K) was determined to be 7.89. When BAR is less than 7.89, the mortality rate shows a relatively obvious increasing trend with the increase of BAR, and the HR is 1.38 (95% confidence interval 0.99–1.92, *p* = 0.0574). When BAR is greater than 7.89, the mortality rate shows a relatively gentle increasing trend with the increase of BAR, and the HR is 1.02 (95% confidence interval 1.00–1.04, *p* = 0.0255). The result of the log-likelihood ratio test (LRT test) shows that *p* = 0.047, indicating that Model II is significantly different from the linear analysis Model I, which strongly confirms the non-linear relationship between BAR and 28-day all-cause mortality, and shows different curve change characteristics on both sides of the turning point.

**Table 2:** Threshold effect analysis of the BAR and 28-day-all-cause mortality.

Measure	HR (95% CI)	*p* value
Model I		
One line effect	1.02 (1.01, 1.04)	0.0038
Model II		
Turning point (K)	7.89	
BAR < K	1.38 (0.99, 1.92)	0.0574
BAR > K	1.02 (1.00, 1.04)	0.0255
*p* value for LRT test*	0.047	

Data were presented as HR (95% CI) *p* value; Model I, linear analysis; Model II, non-linear analysis. Adjusted for Gender, Age (years), BMI(Kg/m^2^), COPD, Diabetes, Arrhythmia, MAP Mean arterial pressure (mmHg), Heart rate (/min), Temperature, AST aspartate transaminase (U/L), total protein (g/dl), SOFA score, GCS score. LRT logarithm likelihood ratio test.

*P < 0.05 indicates that model II is significantly different from Model I.

### Kaplan–meier curves

**Figure 3:**
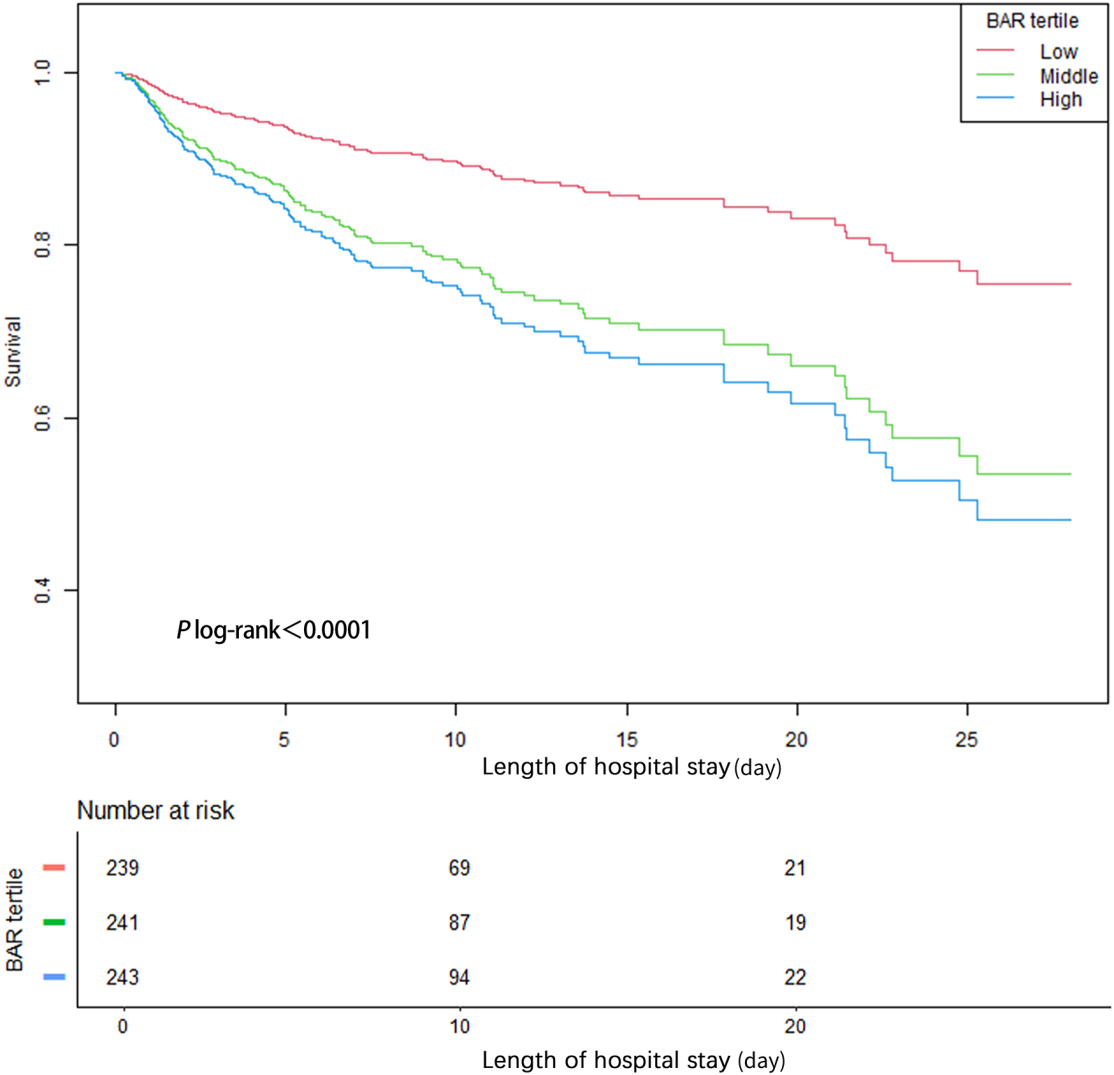
Kaplan–Meier curves.

We used Kaplan–Meier method to conduct survival analysis of BAR levels in the three groups of patients, and drew corresponding survival curves (Figure 3). Survival rates significantly differed across BAR tertiles. The in-hospital survival rate of patients in the high BAR group was significantly lower than that in the other two groups. This finding suggests that high BAR levels are strongly associated with a poorer survival prognosis, suggesting that these patients face a higher risk of death during their hospitalization. Survival curve patterns reflected dynamic changes in patient outcomes during hospitalization. These results highlight the importance of BAR as a potential prognostic biomarker to help healthcare professionals better identify high-risk patients and implement timely interventions to improve their overall survival.

### Multivariable Cox regression analysis

We constructed three multivariate Cox regression models to assess the independent effect of BAR on in-hospital mortality (multivariate Cox regression model). The hazard ratios (HR) and 95% confidence intervals (CI) are shown in Table 3. BAR was analyzed as a continuous variable. In the unadjusted model, for each 1-unit increase in BAR, the 28-day mortality increased by 3% [HR 1.03 95% CI (1.01, 1.04) *p* < 0.0001]. In Model 1, for each 1-unit increase in BAR, the 28-day mortality increased by 3% [HR 1.03 95% CI (1.02, 1.04) *p* < 0.0001]. In Model 3, for each 1-unit increase in BAR, the 28-day mortality increased by 2% [HR 1.02 95% CI (1.01, 1.04) *p* = 0.0038]. The HRs were stable in all unadjusted and adjusted models. For further sensitivity analysis, we transformed the continuous variable BAR into a three-group variable for analysis, using the first group (T1 group) as the baseline reference. In Model 3, the 28-day all-cause mortality in the third group (T3 group) increased by 87% compared to the first group (T1 group) [HR 1.87 95% CI (1.09, 3.19) *p* = 0.023]. In the remaining models, the 28-day mortality in the third group (T3 group) increased significantly, and the increasing trend results were basically stable in both the unadjusted model and the three adjusted models.

**Table 3:** Multivariable Cox regression analysis.

Variable	Unadjusted		Adjust I		Adjust II		Adjust III	
	HR 95% CI	*p* value	HR 95% CI	*p* value	HR 95% CI	*p* value	HR 95% CI	*p* value
BAR	1.03 (1.01, 1.04)	<0.0001	1.03 (1.02, 1.04)	<0.0001	1.03 (1.01, 1.04)	<0.0001	1.02 (1.01, 1.04)	0.0038
BAR tertile								
T1 (0.2–2.3)	1		1		1		1	
T2 (2.3–2.9)	2.24 (1.39, 3.61)	0.001	2.06 (1.27, 3.34)	0.0034	1.99 (1.22, 3.24)	0.0058	1.62 (0.96, 2.75)	0.071
T3 (2.9–4.9)	2.61 (1.64, 4.16)	<0.0001	2.47 (1.54, 3.96)	0.0002	2.32 (1.44, 3.76)	0.0006	1.87 (1.09, 3.19)	0.023
*p* for trend		0.0002		0.0005		0.0044		0.0464

Adjust I model adjusts for: Gender, Age (years).

Adjust II model adjusts for: Gender, Age (years), BMI(Kg/m^2^), COPD, Diabetes, Arrhythmia, MAP Mean arterial pressure (mmHg), Heart rate (/min), Temperature, AST aspartate transaminase (U/L), total protein (g/dl).

Adjust III model adjusts for: Gender, Age (years), BMI(Kg/m^2^), COPD, Diabetes, Arrhythmia, MAP Mean arterial pressure (mmHg), Heart rate (/min), Temperature, AST aspartate transaminase (U/L), total protein (g/dl), SOFA score, GCS score.

### Subgroup analyses

**Figure 4:**
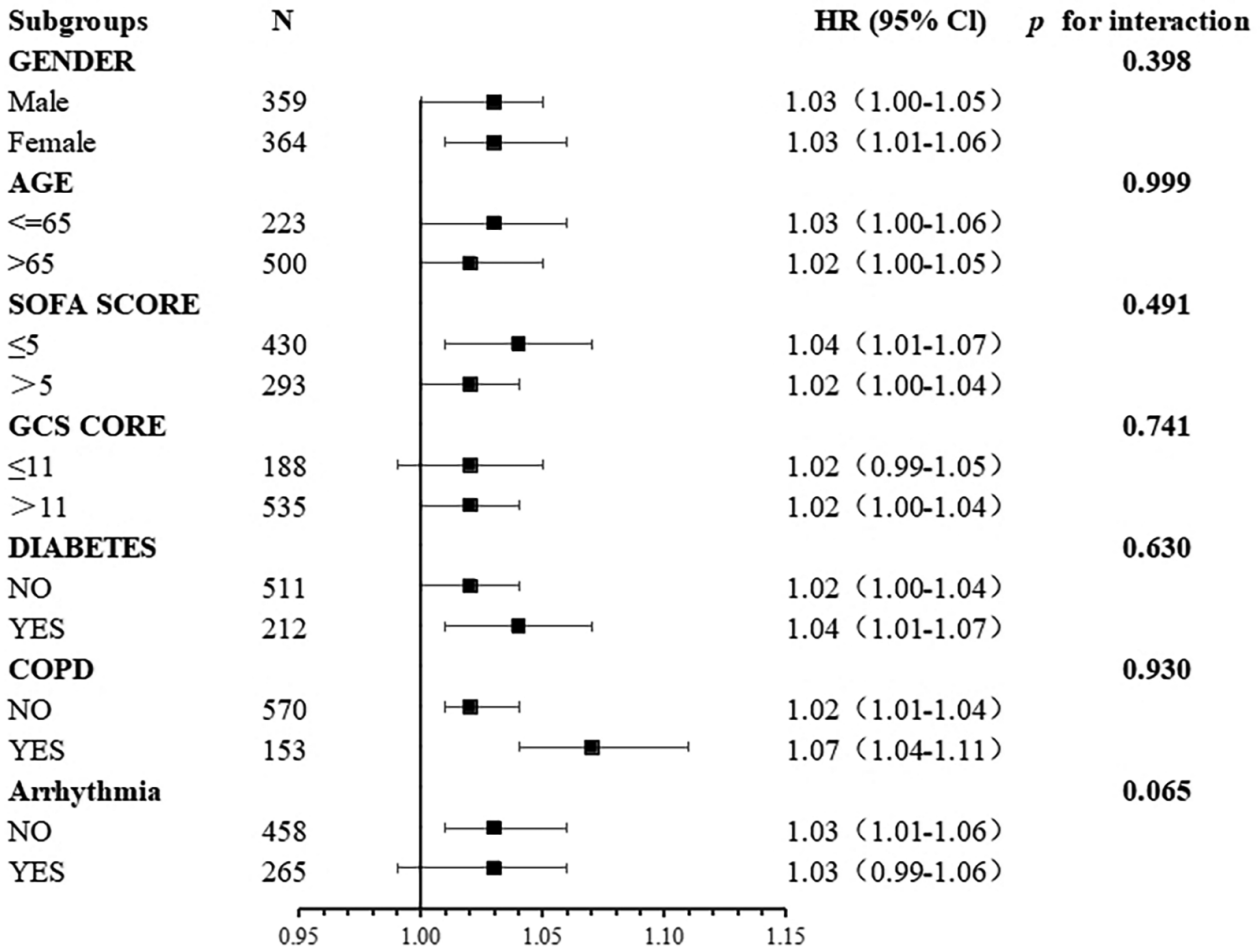
Association between BAR and the risk of all-cause mortality at 28 days in different subgroups.

Subgroup analyses were conducted to test the stability of the relationship between BAR and the risk of all-cause mortality at 28 days (Figure 4). The primary outcome was particularly stable in all subgroups.

The E-value quantifies the minimum strength of association that an unmeasured confounder would need to have with both the exposure and outcome to nullify the observed association. In this analysis, the exposure is BAR, and the outcome is mortality, with a reported hazard ratio (HR) of 1.02. We found that the relative risk (RR) associated with exposure to the unmeasured confounder is 1.22, while the RR between this confounder and mortality is 1.08.The calculated E-value for this study is 1.13, suggesting that if we assume there is no true association between BAR and mortality (i.e., HR = 1), an unmeasured confounder would need to exhibit a minimum association of HR ≥ 1.13 with both BAR and mortality to potentially nullify the observed relationship. This indicates that our results are relatively robust; only unmeasured confounders with strong associations (HR ≥ 1.13) with both the exposure and outcome could weaken our findings. Given the known confounders already adjusted for in the model, we conclude that our results demonstrate significant resilience against potential unmeasured confounding (23). We further validated the results by excluding values beyond the range of mean ± 3 standard deviations, after excluding subjects with BAR values exceeding the mean ± 3 standard deviations, the study population consisted of 707 individuals. After adjusting for all confounding factors, the association between BAR and mortality was consistent with the preliminary analysis (Supplementary Table S1), with HR1.03 (1.02,1.05) *p* ≤ 0.0001 in the unadjusted model and HR1.03 (1.01,1.05) *p* = 0.0016 in Model 3. To more effectively verify the association between BAR and mortality, propensity score matching (PSM) sensitivity analysis was conducted. After matching, a total of 342 subjects were included for multivariate Cox regression analysis to examine the relationship between BAR and mortality. It was found that this relationship was still robust (Supplementary Table S2). In the unadjusted model, HR1.03 (1.01, 1.06) *p* = 0.0152, and in Model 3, HR1.06 (1.03, 1.09) *p* < 0.0001, indicating that propensity score matching successfully reduced the impact of potential bias and made the mortality between the death and survival groups more balanced in terms of covariates. By observing BAR and conducting multivariate Cox regression analysis on the relationship between BAR and mortality rate, the results showed that the association between BAR and mortality rate before and after matching was consistent with the preliminary analysis, and the impact of BAR on mortality rate was significant.

## Discussion

In this study, a retrospective analysis of patients with chronic heart failure combined with sepsis using a large public eICU database revealed a significant linear positive correlation between BAR and 28-day in-hospital mortality. Multiple regression analysis showed a significant increase in 28-day mortality for each 1-unit increase in BAR (HR 1.02, *p* = 0.0038). After dividing BAR into three groups, the highest group had a significantly higher mortality rate than the lowest group (HR 1.87, *p* = 0.023), and the survival curves consistently supported this finding. In addition, subgroup analyses showed that the relationship between BAR and 28-day mortality remained stable across strata of age, gender, COPD, Arrhythmia, GSC score, SOFA score and diabetes.

Both sepsis and chronic heart failure are common high-risk conditions in the intensive care unit (ICU), and patients with chronic heart failure combined with sepsis are also at high risk of death in the ICU (24–27). This study might be the pioneer research exploring the connection between BAR and the acute-phase prognosis of patients with chronic heart failure accompanied by sepsis. It reveals that an elevated BAR level is associated with the acute-phase death risk in such patients and that BAR serves as an independent factor related to the death risk of these patients in the intensive care unit.

Previous studies have shown that elevated BUN levels and hypoalbuminemia are independent risk factors in patients with heart failure and have also confirmed that elevated BUN levels and hypoalbuminemia are independent risk factors in patients with sepsis (28, 29). In the study by Gerasimos Filippatos et al, BUN was a predictor of poor prognosis in patients with chronic heart failure, and the results showed that mortality increased within the baseline BUN quartiles in the included chronic heart failure population. Deaths occurred in 0 (0%), 3 (4.0%), 7 (9.3%), and 10 (14.3%) patients in quartiles 1, 2, 3, and 4, respectively (*p* < 0.001). Baseline BUN was also associated with a composite index of death or hospitalization for heart failure, with 7 (8.6%), 14 (18.4%), 16 (21.3%), and 21 (30.0%) patients experiencing this composite endpoint in quartiles 1, 2, 3, and 4, respectively (*p* *=* 0.005), and high BUN levels reflecting poor levels of cardiac function (30). Elevated blood urea nitrogen levels may reflect the adaptive response of the kidneys to changes in the cardiovascular system in patients with heart failure. When cardiac function declines, it leads to renal ischemia and hypoxia, which in turn leads to a decrease in glomerular filtration rate and accumulation of urea nitrogen in the blood (31). High blood urea nitrogen levels may reflect disturbed heart-kidney interaction, which in turn affects the severity and prognosis of heart failure (32). BUN is also an important indicator for evaluating the severity of sepsis in the study by Xu Li et al. The 30-day mortality rate in septic patients increased with increasing BUN levels, and this association differed at the inflection point of 41.1 mg/dl. When BUN < 41.1 mg/dl, each 10 mg/dl increase in BUN was associated with a 29.8% increase in 30-day mortality in patients with sepsis (HR = 1.298; 95% CI: 1.224–1.376; *p* < 0.001); however, for BUN ≥ 41.1 mg/dl, each 10 mg/dl increase in BUN, 30-day mortality increased by only 4.5% (HR = 1.045; 95% CI: 1.016–1.075; *p* *=* 0.002) (29). In the severe infectious state of sepsis, the patient's body is severely affected by the inflammatory response, leading to the development of a systemic inflammatory response syndrome (33). During this process, the release of inflammatory factors and the production of toxins can affect the function of several organ systems, including the kidneys. Systemic vasodilatation, tissue edema, and hypotension caused by the inflammatory response can lead to renal ischemia and hypoxia, which in severe cases can cause acute kidney injury (AKI). Sepsis leads to a systemic inflammatory response syndrome, where damage to the vascular endothelium leads to vasodilation and blood leakage, which in turn leads to renal hypoperfusion and ischemia (33). Renal ischemia can affect glomerular filtration and reduce the excretion of metabolites such as urea, leading to elevated blood urea nitrogen (34).

Hypoalbuminemia is a common presentation in patients with chronic heart failure and is strongly associated with prognosis. In a study by Israel Gotsman et al, 5,779 patients with heart failure were included with a mean follow-up of 576 days. The results showed that the median serum albumin was 4.0 g/dl, and hypoalbuminemia was present in 12% of the patients (<3.5 g/dl). Cox regression analysis showed that a decrease in albumin quartiles significantly increased the risk of death: the lowest quartile risk ratio was 5.74 (95% CI: 4.08–8.07, *p* < 0.001). Meanwhile, reduced albumin was identified as an independent predictor of increased mortality (HR 2.58, 95% CI: 2.12–3.14, *p* < 0.001) and was associated with increased cardiac-related hospitalizations. Studies have shown that low albumin levels can accelerate the progression of heart failure by causing oxidative stress and endothelial dysfunction (35). Albumin is primarily synthesized by the liver, and patients with chronic heart failure often have liver dysfunction or liver damage (36). The liver's ability to synthesize albumin is diminished, resulting in decreased serum albumin levels. Patients with heart failure often experience edema and protein loss, leading to increased albumin loss. Protein loss is primarily caused by factors such as tissue edema, increased intestinal permeability, and inflammatory response (37). Patients with heart failure often have a chronic inflammatory state, and the release of inflammatory factors can affect albumin synthesis and stability, leading to decreased albumin levels. Chronic heart failure often leads to ischemia and hypoxia in the digestive system, resulting in decreased digestion and absorption and other functions (38). Patients with loss of appetite and weakened digestion and absorption are prone to malnutrition, leading to decreased albumin synthesis. Heart failure patients have metabolic disorders, which can lead to increased energy consumption and protein metabolism disorders, affecting albumin levels (39). Overall, the mechanisms of albumin reduction in heart failure patients are multifaceted. Albumin is closely related to sepsis, and albumin plays an important role in the onset, progression, and prognosis of sepsis (40). In a study conducted by Si Hyoung Lee et al., a total of 493 patients with sepsis were analyzed, with 140 (28.4%) fatalities recorded. The findings indicated that albumin concentrations were significantly lower in non-survivors compared to survivors, measuring 3.3 ± 0.6 mg/dl and 2.8 ± 0.6 mg/dl, respectively. Furthermore, the mortality rate was significantly higher in the hypoalbuminemic group at 41.2% compared to 10.3% in the normoalbuminemic group (*p* < 0.01). And the mortality rate was higher in the hypoalbuminemic group than in the normoalbuminemic group (41.2% and 10.3%, respectively, *p* < 0.01). In Cox analysis, hypoalbuminemia was associated with a 3.8-fold increased risk of death during the 28-day follow-up (hazard ratio, 3.83; 95% CI, 2.22–6.59). The AUC for albumin concentration was 0.73 (95% CI, 0.69–0.78), which was comparable to the APACHE II score (0.77; 95% CI, 0.73–0.81) (41). During the onset of sepsis, the inflammatory response of the body leads to a significant decrease in albumin levels, which is considered one of the manifestations of the inflammatory state that septic patients are in (42). Albumin is an important protein for the maintenance of plasma osmolality and is essential for maintaining blood volume homeostasis (43). Patients with sepsis are prone to hypovolemia and edema due to increased vascular permeability and altered plasma osmolality, which affects organ function. Patients with sepsis have a faster metabolic rate and require more nutritional support to maintain basic physiologic activities and restore damaged tissues (44). Albumin is an important nutrient protein that helps maintain normal body function and tissue repair (45). Decreased plasma albumin levels may affect the nutritional status and recovery of patients with sepsis (46).

BAR makes up for the limitations in indicating the association between poor prognosis and the conditions of high serum urea nitrogen and low albuminemia. It provides a more comprehensive view of the relationship among these factors in diseases. Compared to albumin or BUN, BAR demonstrates a stronger correlation with improved acute-phase survival rates in patients with chronic heart failure complicated by sepsis, and may offer relevant implications for both treatment protocol selection and clinical outcomes. In this study we used BAR as a research subject, BAR is a composite indicator combining blood urea nitrogen and albumin, both serum urea nitrogen and albumin are relatively easy to obtain biochemical indicators in clinical practice, which is one of the factors why we chose BAR as a research subject. The possible mechanism of the relationship between BAR and poor prognosis can be both BUN and albumin as described above explanation. In patients with chronic heart failure combined with sepsis, hypoalbuminemia with high serum urea nitrogen is often present. BUN is an important indicator of renal function. Chronic heart failure leads to a decrease in cardiac output, which affects the perfusion and excretory function of the kidneys, which in turn increases the level of urea nitrogen in the blood. Albumin is an important indicator of the patient's nutritional status. Hepatic insufficiency due to chronic heart failure and sepsis often leads to reduced protein synthesis and malnutrition, resulting in a decrease in albumin levels, and sepsis systemic inflammatory response syndrome leads to metabolic disorders and hepatic anabolic dysfunction, which increases protein catabolism and thus reduces albumin levels. At the same time, the inflammatory response makes renal function impaired, exacerbating the elevated BUN. This inflammatory state may be closely related to the patient's prognosis. Patients with heart failure often have fluid retention and electrolyte imbalance, and BUN levels may be affected by changes in fluid status. In conclusion, BAR levels in patients with chronic heart failure combined with sepsis are closely associated with hypoalbuminemia and high serum urea nitrogen in patients with this type of complex disease. In the present study, the high risk of death in patients with high BAR levels may be highly associated with renal insufficiency, hepatic insufficiency, poor nutritional status, systemic inflammatory response, fluid management, and poor fluid status.

According to the research results, when facing patients with chronic heart failure with sepsis, clinicians should take multi-dimensional measures. Incorporate BAR into routine monitoring, especially closely monitor those in the highest BAR tertile. Consider factors like cardiac function and infection severity to form personalized treatment plans and enhance support. For hypoalbuminemia patients, carefully supplement albumin after assessing serum albumin and hemodynamics, adjust diet to high—calorie, vitamin—rich, sodium—controlled meals, and use enteral or parenteral nutrition if digestion is impaired. As BAR correlates with mortality, control blood urea nitrogen by optimizing renal function, like rational diuretic use and careful nephrotoxic drug control, and use renal replacement therapy when needed. BAR guides early sepsis management, prompting more aggressive anti—infection for high—BAR patients, but it must be combined with other clinical data to avoid over—treatment and improve patient outcomes.

Our study has made an effort to explore the relationship between BAR and 28-day in-hospital mortality in patients with chronic heart failure and sepsis through subgroup analyses based on several key covariates. However, we are aware that there may exist subphenotypes that could potentially modify this relationship and are not fully captured by our current approach. Subphenotypes identified in higher dimensional space might display differences in the association strength between variables of interest. In our study, although we have stratified our analysis based on factors such as gender, age, COPD, diabetes, arrhythmia, SOFA score, and GCS score, there could still be underlying heterogeneity that remains unexplored. For example, patients with similar demographic and clinical characteristics within a particular subgroup might still have different pathophysiological processes at play, which could in turn affect the role of BAR in predicting mortality. In patients with chronic heart failure (CHF) and sepsis, mortality risk is influenced by several clinical factors. Age plays a critical role, as elderly patients (>65 years) exhibit reduced physiological resilience and impaired immune responses, exacerbating both cardiac dysfunction and sepsis progression. Comorbidities further amplify risk: COPD impairs pulmonary gas exchange and increases cardiac strain, while diabetes promotes bacterial proliferation and compromises vascular integrity. Disease severity scores provide prognostic insights—elevated SOFA scores indicate multi-organ dysfunction, and low GCS scores reflect neurological impairment, both strongly correlating with mortality. Timely interventions are pivotal; early fluid resuscitation, targeted antimicrobial therapy, and optimized CHF management significantly improve outcomes, whereas delays or inappropriate treatments may accelerate clinical deterioration.

This study discovered that elevated BAR levels are closely associated with an increased risk of 28—day mortality in patients with chronic heart failure combined with sepsis. In this patient population, BAR emerges as an independent risk factor linked to acute—phase mortality. Therefore, it can be regarded as a novel biomarker that has a strong association with the risk of mortality. BAR has an important value for clinical application, and it can be used for early screening and risk assessment, which can help physicians to perform stratified management and personalized treatment. Meanwhile, by dynamically monitoring BAR levels, physicians can adjust treatment strategies and implement early interventions to reduce the risk of death and enhance patients' and their families' understanding of the prognosis of the disease, thus improving clinical outcomes.

## Conclusion

High levels of BAR are independently associated with the 28—day mortality risk in patients with chronic heart failure complicated by sepsis in the intensive care unit. There is a significant correlation between BAR and the prognosis of these patients. BAR can be regarded as a convenient and efficient biomarker closely related to the poor prognosis of patients with chronic heart failure complicated by sepsis. However, the underlying mechanisms behind the association of BAR with the outcome and the implications for clinical treatment still demand further in—depth investigation.

## Limitation

One significant limitation of our study, being a retrospective one, is that it can only establish associations rather than causal relationships. The causal connection between the Blood Urea Nitrogen to Serum Albumin Ratio (BAR) and 28—day all—cause mortality cannot be definitively determined. This is a notable shortcoming considering the implications of this research. Future longitudinal or experimental studies are essential as they would help clarify these causal relationships, enabling a more profound understanding of the underlying mechanisms.

Another major concern is the potential presence of unmeasured confounding factors. Even though we've made efforts to incorporate a wide range of confounding variables such as gender, age, BMI, various disease scores, and laboratory test results into our analysis, there might still be other unaccounted—for elements. For instance, BNP, Troponin, socioeconomic status, lifestyle factors, fluid management, nutritional support, specific treatment interventions, and specific genetic polymorphisms could all impact both the BAR levels and the risk of mortality in these patients. Although we've adjusted for numerous possible confounders, it's arduous to completely eliminate the potential influence of unmeasured variables on our results. To address this issue, we have employed appropriate statistical approaches, such as multivariable Cox regression models. However, some uncertainty still remains. To quantify the potential impact of unmeasured confounders, we conducted an E—value sensitivity analysis. The results indicated that an unmeasured confounder was unlikely to influence the correlation between BAR and mortality within the study population.

Data—related limitations also exist. Some patients had missing data for certain variables, like albumin or blood urea nitrogen, which reduced the sample size available for analysis and might have introduced bias,the potential impact on the validity of our results cannot be overlooked. Additionally, the data source was a specific eICU database, and the generalizability of our findings to other populations or healthcare settings may be restricted. The characteristics of the patients in this database might not comprehensively represent the entire spectrum of patients with chronic heart failure and sepsis in different regions or hospitals.

Finally, the relatively small sample size of 723 patients, while sufficient to detect some significant associations, may not offer enough statistical power to thoroughly explore more intricate relationships or subgroup differences. Larger sample sizes in future studies would enhance the reliability and robustness of the results and allow for more detailed subgroup analyses, thereby facilitating a better understanding of the heterogeneity within the patient population.

## Data Availability

The original contributions presented in the study are included in the article/Supplementary Material, further inquiries can be directed to the corresponding author/s.
