# Supplementary material for: The association between blood urea nitrogen to serum albumin ratio and 28 day in-hospital mortality in patients with chronic heart failure and sepsis: a pilot retrospective study
Source: Front Cardiovasc Med. 2025 Mar 4;12:1491331. doi: 10.3389/fcvm.2025.1491331 (PMC11914131; doi:10.3389/fcvm.2025.1491331)
Supplement: Supplementary file 1 [file Datasheet1.pdf]

Supplementary Information

1) E-value analysis

The E-value quantifies the minimum strength of association that an unmeasured confounder would need to have with both the exposure and outcome to fully explain away the observed association. In this analysis, the exposure is the BAR, and the outcome is mortality, with a reported hazard ratio (HR) of 1.02. We found that the relative risk (RR) associated with exposure to the unmeasured confounder is 1.22, while the RR between this unmeasured confounder and mortality is 1.08.The E-value for this study is 1.13, indicating that if we assume there is no true association between BAR and mortality (i.e., HR=1), an unmeasured confounder would need to demonstrate a minimum association of HR ≥ 1.13 with both BAR and mortality to potentially nullify the observed relationship. This analysis suggests that our results are relatively robust; only unmeasured confounders with strong associations (HR ≥ 1.13) with both the exposure and outcome could undermine our findings. Given the known confounders already adjusted for in the model, we conclude that our results have substantial resilience against potential unmeasured confounding.

2) Table S1. Excluding values in BAR that are more than three standard deviations above or below the average, multivariable Cox regression analysis. (N=707),

Figure S1. Propensity score based on linear model

Table S2. After excluding values in BAR that are more than three standard deviations above or below the average, 707 subjects were included. After propensity score matching,342 subjects were included, followed by multivariable Cox regression analysis(N=342).

TableS1 Excluding values in BAR that are more than three standard deviations above or below the average, multivariable Cox regression analysis.

Variable	Unadjusted		Adjust I		Adjust II		Adjust III	
	HR 95%CI	*p* value	HR 95%CI	*p* value	HR 95%CI	*p* value	HR 95%CI	*p* value
**BAR**	1.03 (1.02, 1.05)	<0.0001	1.03 (1.02, 1.05)	<0.0001	1.04 (1.02,1.05)	0.0002	1.03 (1.01, 1.05)	0.0016
**BAR tertile**								
T1(0.2-2.3)	1	1	1		1		1	
T2(2.3-2.9)	2.11(1.30,3.43)	0.0024	1.96 (1.21, 3.20)	0.0065	1.85 (1.13,3.20)	0.0146	1.52 (0.89, 2.59)	0.1219
T3(2.9-4.9)	2.54(1.59,4.07)	<0.0001	2.36 (1.46, 3.79)	0.0004	2.17 (1.33,3.54)	0.0018	1.84 (1.07, 3.16)	0.0270

Adjust I model adjust for: Gender, Age (years).

Adjust II model adjust for: Gender, Age (years), BMI(Kg/m2), COPD, Diabetes, Arrhythmia, MAP Mean arterial pressure (mmHg), Heart rate (/min), Temperature, AST aspartate transaminase (U/L), total protein (g/dL).

Adjust IIImodel adjust for: Gender, Age (years), BMI(Kg/m2), COPD, Diabetes, Arrhythmia, MAP Mean arterial pressure (mmHg), Heart rate (/min), Temperature, AST aspartate transaminase (U/L), total protein (g/dL), SOFA score, GCS score.

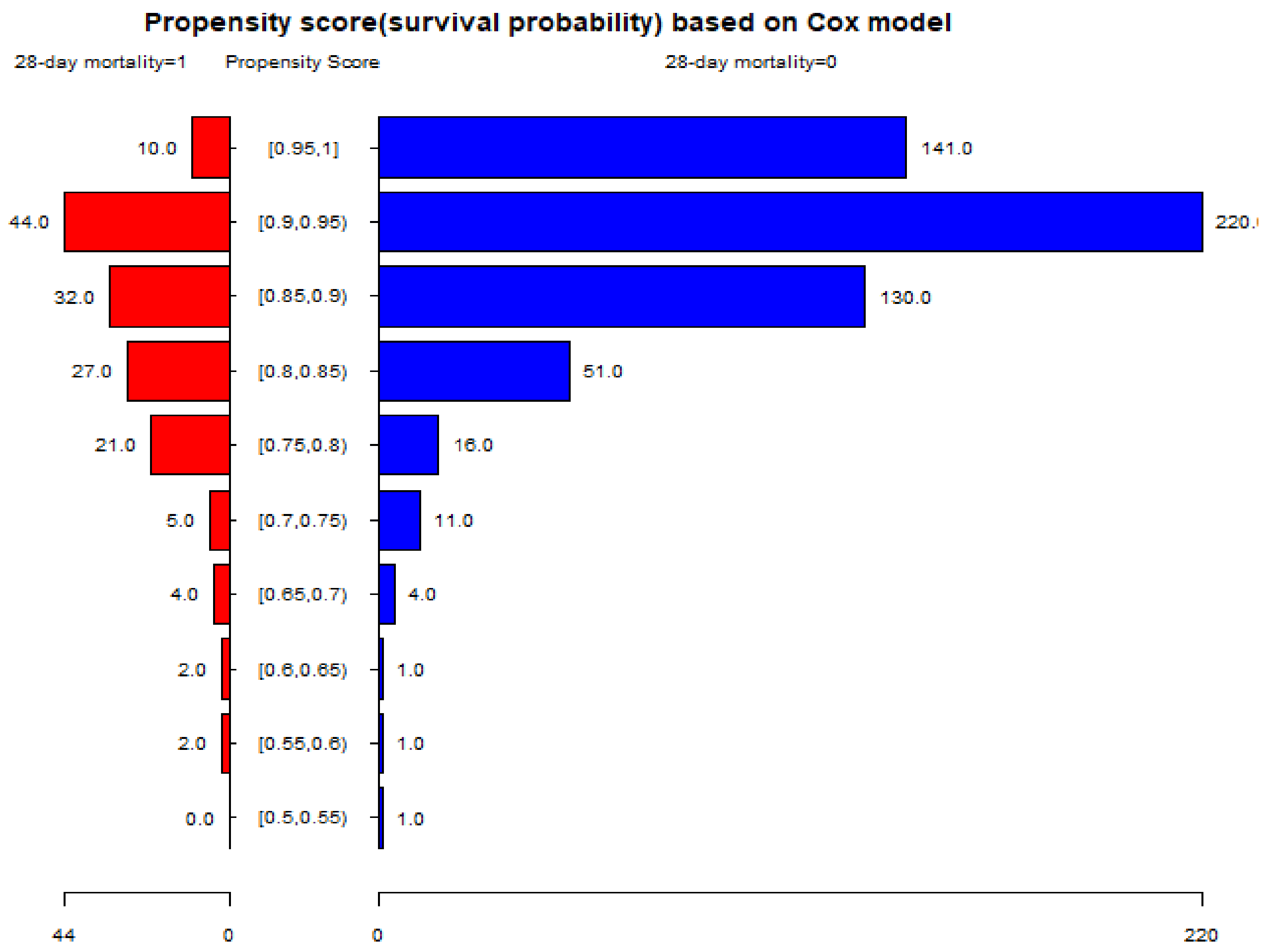

Figure S1. Propensity score based on linear model

TableS2 Multivariable Cox regression analysis after propensity score matching.

Variable	Unadjusted		Adjust I		Adjust II		Adjust III	
	HR 95%CI	*p* value	HR 95%CI	*p* value	HR 95%CI	*p* value	HR 95%CI	*p* value
**BAR**	1.03 (1.01, 1.06)	0.0152	1.03 (1.01, 1.06)	0.0013	1.05 (1.02,1.07)	0.0005	1.06 (1.03, 1.09)	<0.0001
**BAR tertile**								
T1(0.2-2.3)	1		1		1		1	
T2(2.3-2.9)	2.47 (1.18, 5.15)	0.0162	2.44(1.17,5.11)	0.0178	2.53(1.18,5.38)	0.0165	3.83(1.64,8.95)	0.0019
T3(2.9-4.9)	2.47 (1.16, 5.25)	0.0033	2.46(1.15,5.27)	0.0203	2.77(1.26,6.10)	0.0111	4.86(1.99,11.87)	0.0005

Adjust I model adjust for: Gender, Age (years).

Adjust II model adjust for: Gender, Age (years), BMI(Kg/m2), COPD, Diabetes, Arrhythmia, MAP Mean arterial pressure (mmHg), Heart rate (/min), Temperature, AST aspartate transaminase (U/L), total protein (g/dL).

Adjust IIImodel adjust for: Gender, Age (years), BMI(Kg/m2), COPD, Diabetes, Arrhythmia, MAP Mean arterial pressure (mmHg), Heart rate (/min), Temperature, AST aspartate transaminase (U/L), total protein (g/dL), SOFA score, GCS score.
